# Extracellular matrix and cytochrome P450 gene expression can distinguish steatohepatitis from steatosis in mice

**DOI:** 10.1111/jcmm.12328

**Published:** 2014-06-09

**Authors:** Ewa E Hennig, Michal Mikula, Krzysztof Goryca, Agnieszka Paziewska, Joanna Ledwon, Monika Nesteruk, Marek Woszczynski, Bozena Walewska-Zielecka, Kazimiera Pysniak, Jerzy Ostrowski

**Affiliations:** a Department of Gastroenterology and Hepatology, Medical Center for Postgraduate EducationWarsaw, Poland; b Department of Genetics, Maria Sklodowska-Curie Memorial Cancer Center and Institute of OncologyWarsaw, Poland; c Department of Public Health, Health Sciences Faculty, Warsaw Medical UniversityWarsaw, Poland

**Keywords:** steatosis, steatohepatitis, NAFLD, NASH, obesity, transcriptomics, liver, mouse model, extracellular matrix, cytochrome P450

## Abstract

One of the main questions regarding nonalcoholic fatty liver disease is the molecular background of the transition from simple steatosis (SS) to the inflammatory and fibrogenic condition of steatohepatitis (NASH). We examined the gene expression changes during progression from histologically normal liver to SS and NASH in models of obesity caused by hyperphagia or a high-fat diet. Microarray-based analysis revealed that the expression of 1445 and 264 probe sets was changed exclusively in SS and NASH samples, respectively, and 1577 probe sets were commonly altered in SS and NASH samples. Functional annotations indicated that transcriptome alterations that were common for NASH and SS, as well as exclusive for NASH, involved extracellular matrix (ECM)-related processes, although they differed in the type of matrix structure change. The expression of 80 genes was significantly changed in all three comparisons: SS *versus* control, NASH *versus* control and NASH *versus* SS. Of these genes, epithelial membrane protein 1, IKBKB interacting protein and decorin were progressively up-regulated in both SS and NASH compared to normal tissue. The molecular context of interactions of encoded 80 proteins revealed that they are highly interconnected and significantly enriched for processes involving metabolism by cytochrome P450. Validation of 10 selected mRNAs encoding genes related to ECM and cytochrome P450 with quantitative RT-PCR analysis showed consistent changes in their expression during NASH development. The expression profile of these genes has the potential to distinguish NASH from SS and normal tissue and may possibly be beneficial in the clinical diagnosis of NASH.

## Introduction

Abnormal or excessive fat accumulation in hepatocytes reflects the hepatic manifestation of metabolic syndrome, termed nonalcoholic fatty liver disease (NAFLD) [1]. NAFLD is associated with the accumulation of visceral fat, hyperlipidaemia and insulin resistance (IR), which affects insulin-mediated glucose disposal and gluconeogenesis in muscle, as well as fatty acid synthesis in the liver [2–5]. NAFLD has a worldwide distribution, with a prevalence of 17–33% in the general population [6]. It is composed of a spectrum of liver disorders, ranging from simple steatosis (SS), through steatohepatitis (NASH), to cirrhosis and even hepatocellular carcinoma [7]. Although liver steatosis can result from various pathological conditions underlying lipid metabolism, it is largely related to obesity [8]; more than 75% of obese adults and 50% of obese children can be affected by excessive triglyceride accumulation in the liver [9,10]. The increasing incidence of obesity, combined with a caloric-rich diet and a low level of physical activity, makes NAFLD a significant health problem.

Because animal models of obesity reflect the behavioural and metabolic features that predispose humans to chronic caloric overconsumption, they appear to be a valuable tool to study the mechanisms underlying NAFLD pathogenesis and the progression to NASH. Animal obesity can be achieved by forced feeding, administration of high-caloric diets, genetic modification of the feeding behaviour, or a combination of these approaches [11]. Prolonged feeding with a high-fat diet (HFD) or the disruption of leptin signalling in leptin-deficient (ob/ob) and leptin receptor-deficient (db/db) hyperphagic mice results in hepatic steatosis accompanied by obesity, IR, changes in lipid and glucose homoeostasis and oxidative stress [12,13].

NAFLD-associated phenotypes, both in humans and animal models of obesity, can be studied by using high-throughput genomic methods that allow the profiling of transcripts on a global scale. Microarray-based studies revealed altered expression of numerous genes involved in hepatic glucose and lipid metabolism, insulin signalling, inflammation, coagulation, cell adhesion, oxygen stress and the activity of chaperones [14–17]. The up-regulation of hepatic genes related to cell survival and liver regeneration and the down-regulation of genes involved in defence mechanisms against oxidative stress were identified during the early stages of NAFLD. In turn, the up-regulation of genes involved in hepatic stellate cell (HSC) activation and detoxification pathways, which are modulated by the expression of tumour necrosis factor (TNF)-α, interleukin (IL)-6 and interferon (IFN)-γ, was noticeable at the NASH stage [14,15,18–21].

However, despite the numerous studies that have been performed until now, the mechanisms underlying the progression from SS to NASH remain incompletely understood. As recently reported, in humans, NASH, but not SS, exhibits a molecular profile that is characteristic for the processes relevant to malignant tumours, suggesting that it may reflect a premalignant state of liver disease already at the pre-cirrhotic stage [10]. In this study, we focused on hepatic gene expression profiling during the progression from a histologically normal liver to SS and to NASH in mouse models of obesity caused by hyperphagia or HFD. Comprehensive transcriptomic analysis indicated that extracellular matrix (ECM)-related processes were the most consistently altered during the progression to NASH in the mouse liver.

## Material and methods

### Animals

Five-week-old male wild-type (wt) C57BL/6J mice and mice homozygous for the leptin gene mutation B6.V-*Lep*^*ob*^/J (ob/ob) or the leptin receptor gene mutation B6.BKS(D)-*Lepr*^*db*^/J (db/db) were purchased from The Jackson Laboratory, Bar Harbor, ME, USA. Throughout this study, the mice were housed under temperature (21 ± 2°C) and humidity (55 ± 10%) controlled conditions, with a 12 hrs light/dark cycle and *ad libitum* access to food and water. Experimental protocols were approved by The 2nd Local Ethical Committee for Animal Research in Warsaw, Poland.

### Experimental design

During 1 week of adaptation, all mice were fed a normal diet (ND; 10% of calories from fat) containing 19.2% protein, 67.3% carbohydrate and 4.3% fat (D12450B; Research Diets, New Brunswick, NJ, USA). At 6 weeks of age, 24 wt C57BL/6J mice were further fed a ND (control group), while another 24 wt C57BL/6J mice were fed a HFD (HFD group; 60% of calories from fat) containing 26.2% protein, 26.3% carbohydrate, and 34.9% fat (D12492; Research Diets). Twenty four ob/ob and 24 db/db mice were fed a ND throughout the entire experiment. Before sacrifice, half of the mice in each group were deprived of food for a period of 18 hrs (from 3 pm until 9 am next day). At either 16 weeks or 48 weeks of age, the mice were weighed and killed, followed by the rapid collection of blood and liver tissues.

### Histopathology

Fragments of fresh liver tissue were formalin-fixed and paraffin-embedded for histopathological examination. Then, the tissue samples were routinely processed; the slides were cut and stained with haematoxylin and eosin. Histological features, including the degree of steatosis, inflammation and hepatocyte ballooning, were assessed by an experienced pathologist in a blinded fashion and NAFLD activity score (NAS) was calculated (Table S1) according to the modified Brunt criteria for histological diagnosis of NASH [22,23].

### Serum biochemical analyses

Serum glucose, cholesterol, triglyceride, alanine aminotransferase (ALT), aspartate aminotransferase (AST), and alkaline phosphatase (ALKP) levels were determined by spectrometry by using a VITROS analyzer in a EKTAchem DT-60-II system (modules DT, DTE, DTSC) and sets of ready-to-use slides (Ortho-Clinical Diagnostics, Johnson & Johnson, Raritan, NJ, USA). Serum insulin concentrations were determined by using a commercially available rat/mouse insulin ELISA kit (Millipore Corp., Billerica, MA, USA).

### mRNA extraction

Total RNA was individually isolated from each liver sample by using the RNeasy Plus Mini Kit (Qiagen, Hilden, Germany), followed by on-column DNAse I digestion. The quality of the RNA samples was determined by using an Agilent 2100 Bioanalyzer (Agilent Technologies, Santa Clara, CA, USA); the samples used for microarray analysis displayed distinct peaks corresponding to intact 28S and 18S ribosomal RNA. Equal amounts of RNA (500 ng) were combined from two randomly selected animals belonging to the same group (defined obesity model, age and feeding status). This process was performed for the six animals in each group, resulting in three biological replicates of pooled RNA per trait.

### Microarray analyses

The average signal from the MouseRef-8 v2.0 Expression BeadChips (AROS Applied Biotechnology, Aarhus N, Denmark) was quantile normalized with no background correction. All computations were performed with the R 2.15.0 software [24] with the Bioconductor extension [25]. Illumina and the Kyoto Encyclopedia of Genes and Genomes (KEGG) [26] identifiers were mapped to the genes by using the lumiMouseAll.db (version 1.18.0), KEGG.db (version 2.8.0), and lumi (version 2.8.0) packages [27]. The measurements were filtered according to the ratio of the interquartile range (IQR) to the median. Only probes with a ratio greater than 1:8 were selected for analysis. Differentially expressed probe sets were identified by using a *t*-test (Welch variant), followed by the Benjamini-Hochberg *P*-value correction for multiple hypotheses testing. Adjusted *P*-values (*P*_*adj*_) ≤ 0.05 were considered significant. Microarray data were deposited in the Gene Expression Omnibus database under entry GSE43691.

### Quantitative reverse transcriptase PCR (qRT-PCR)

RNA concentrations were determined in 90 individual liver tissue samples by using qRT-PCR, as previously described [28]. The expression of Mrpl36, Hmbs and Mcoln1 was used to generate a normalizing factor with the GeNorm software [29]. The sequences of all primers are listed in Table S2. Differences were evaluated by using a *t*-test and were corrected for multiple hypotheses testing by using the Benjamini-Hochberg procedure. A *P*_*adj*_ ≤ 0.05 was considered significant.

### Functional analyses

The differentially expressed probe sets were functionally annotated according to the pre-defined biological pathways included in the ConsensusPathDB (CPDB) interaction database [30]. The enriched pathway-based sets were selected according to the hypergeometric test, and *P*-values ≤ 0.01 were considered significant. To explore the network of selected proteins and their contribution to the KEGG pathways, we used the STRING (version 9.05) software to construct functional protein-interaction networks and calculate their significance based on aggregated experimental data [31].

### Statistical analyses of biochemical dataset

Statistical analyses were performed with the Mann–Whitney *U*-test, and *P*-values ≤ 0.05 were considered significant. Hierarchical clustering was performed on the scaled data, by using an average method with Euclidean distance as the dissimilarity measure.

## Results

To define the gene expression profile changes during the progression to NASH, we used hepatic samples from young (16-week-old) and old (48-week-old) mice from three obesity models: hyperphagic ob/ob and db/db mice with disruption of leptin signalling and wt C57BL/6J mice fed a HFD. Twelve mice were analysed in each group of a given genotype, age and diet. To determine the effect of fasting on the measured parameters, half of the mice in each group were deprived of food overnight (18 hrs) prior to sacrifice, according to typical mice metabolic study regimens [32].

As we previously reported, the body mass and liver mass of the ob/ob, db/db and HFD-fed mice at the age of 16 and 48 weeks were significantly (*P* < 0.01) higher than those of the ND-fed control mice [33]. Overnight fasting resulted in a significant decrease in the liver weight of all the control mice, as well as the young ob/ob and HFD-fed mice, compared to the non-fasted animals.

### Liver histology

Livers of both 16- and 48-week-old control mice, as well as young HFD-fed mice, were histologically normal (Fig. S1). Significant hepatic fat accumulation was observed in other 59 obese mice, exceeding 65% of hepatocytes in 56 animals; this level of accumulation is considered severe according to stratification of tertiles of steatosis [23]. Young and old ob/ob mice developed both macrovesicular steatosis localized to lobular zone 2 and microvesicular steatosis that prevailed at perivenular and periportal locations. In 48-week-old ob/ob mice, these changes were accompanied by moderate inflammatory activity with no signs of fibrosis (Fig. S1). A number of ballooned hepatocytes were also observed. Young and old db/db mice predominantly displayed severe macrovesicular steatosis, which was found in almost all of the hepatocytes in older mice and was associated with moderate necroinflammatory activity and ballooning. A notable feature of the 48-week-old db/db group was the proliferation of cells resembling biliary epithelium at the periphery of the portal spaces and, to a lesser degree, at centrilobular locations. Livers of 48-week-old HFD-fed mice displayed severe mixed microvesicular and macrovesicular steatoses, accompanied by mild inflammation without fibrosis.

Overnight fasting caused noticeable morphological changes in the liver in all control mice and in the 16-week-old HFD-fed mice and resulted in moderate vacuolization of the hepatocyte cytoplasm, which was observed as the presence of small vacuoles without peripheral displacement of the nucleus (Fig. S1). In all other obese mice, 18 hrs of fasting did not alter the histological pictures of hepatic steatosis.

As is widely accepted, the key histological features of NASH include steatosis, lobular inflammation and hepatocellular ballooning, which are currently used as components of the NAFLD activity scoring systems [22,34]. The presence of fibrosis is not considered a condition required for NASH diagnosis. According to this NAFLD scoring and grading, in our study, 48-week-old genetically modified and HFD-fed obese mice were considered animals that developed NASH (Table S1).

### Serum biochemical measures

Histological observations were verified by biochemical measurements. The serum levels of the biochemical parameters measured at both 16- and 48-week time-points in the non-fasted and fasted mice are shown in Table 1.

**Table 1:** Serum biochemical parameters at 16-week and 48-week time-points

	Fasted				Non-fasted			
		
	ob/ob	db/db	HFD	Control	ob/ob	db/db	HFD	Control
16-weeks								
GLU (mmol/l) median (range)	10.4* (5.5–11.7)	8.0 (2.5–9)	6.7* (6.1–7.4)	3.0 (2–5.8)	6.6 (4–11.4)	6.6 (5.9–7.4)	9.4* (7.3–13.4)	6.8 (5.8–9.4)
INS (ng/ml) median (range)	3.61* (2.54–6.35)	4.66* (2.19–7.46)	0.86* (0.36–1)	0.08 (0.04–0.18)	8.30* (5.74–55.91)	7.91* (4.46–43.23)	4.17* (1.81–10.04)	0.83 (0.5–1.35)
AST (U/l) median (range)	756* (316–1072)	776* (324–2825)	142 (109–185)	158 (129–245)	645* (539–1154)	384* (264–533)	90 (64–199)	120 (66–195)
ALT (U/l) median (range)	672* (288–895)	734* (293–1850)	46* (39–63)	32 (21–40)	598* (490–1258)	354* (275–486)	39 (36–68)	29 (25–67)
ALKP (U/l) median (range)	483* (305–650)	398* (221–470)	75 (61–84)	88 (28–99)	532* (415–674)	350* (279–433)	71* (63–92)	96 (87–114)
CHOL (mmol/l) median (range)	5.63* (5.13–6.08)	5.28* (3.56–6.28)	3.48 (2.84–3.53)	2.83 (2.47–3.7)	5.90* (5.54–7.3)	5.11* (4.74–6.52)	3.74* (3.62–4.12)	3.12 (2.49–3.52)
TRIG (mmol/l) median (range)	1.13* (1.09–1.25)	0.98 (0.79–1.27)	1.15* (1.13–1.39)	1.00 (0.86–1.21)	1.05 (0.88–1.24)	0.80* (0.62–0.91)	1.13 (0.92–1.33)	1.04 (0.97–1.45)
48-weeks								
GLU (mmol/l) median (range)	10.9* (6.9–16.6)	8.0* (3.9–14.3)	7.0* (5.9–12)	3.1 (2.1–4.6)	6.1 (5.4–6.6)	6.7 (5.4–12.1)	7.8 (6.8–9.7)	7.3 (5.8–10.5)
INS (ng/ml) median (range)	4.74* (3.06–6.77)	6.56* (2.74–22.73)	2.80* (1.86–5.14)	0.16 (0.04–0.21)	20.87* (8.07–85.61)	10.26* (4.38–55.36)	32.36* (14.25–50.8)	1.83 (0.21–5.81)
AST (U/l) median (range)	672* (244–1164)	577* (262–3510)	467* (345–580)	190 (117–273)	349* (210–473)	333* (190–579)	313* (231–570)	137 (110–258)
ALT (U/l) median (range)	570* (108–794)	422* (180–2628)	352* (269–537)	28 (16–62)	229* (118–360)	251* (117–393)	245* (190–556)	28 (17–148)
ALKP (U/l) median (range)	344* (152–379)	254* (192–455)	181* (84–282)	103 (82–123)	280* (139–417)	214* (174–294)	122 (111–208)	103 (55–137)
CHOL (mmol/l) median (range)	4.97* (3.53–5.99)	5.36* (4.23–15)	5.22* (4.21–6.66)	3.00 (2.26–3.35)	6.06* (4.74–7.14)	5.80* (3.75–6.89)	5.47* (4.82–6.01)	3.68 (2.57–4.15)
TRIG (mmol/l) median (range)	1.30* (1.19–1.38)	1.07* (0.9–1.55)	1.19* (0.95–1.39)	0.69 (0.55–0.9)	0.98 (0.8–1.14)	0.85 (0.44–1.51)	0.92 (0.79–1.1)	0.74 (0.54–0.95)

* *P* < 0.05, compared with the relevant control group. HFD; high-fat diet fed mice.

#### Serum glucose and insulin concentrations

Because, in contrast to humans, mice are nocturnal feeders, the non-fasting glucose and insulin levels might be considered baseline measures. Among all of the obese mice, the baseline glucose level was significantly higher (*P* = 0.041) in the 16-week-old HFD-fed mice than in the control mice. The overnight-fasted young and old control mice, as well as the young HFD-fed mice, exhibited baseline glucose levels that were significantly suppressed (*P* ≤ 0.006) by 2.3-, 2.4- and 1.4-fold, respectively, while the 48-week-old HFD-fed mice exhibited only a slight, not significant, reduction. In contrast, 18 hrs fasting resulted in an increase in the serum glucose level by 1.8-fold (*P* = 0.005) in the 48-week-old ob/ob mice. In the fasted 16-week-old ob/ob mice and both the young and old db/db mice, the serum glucose concentration was not significantly increased.

The serum baseline insulin concentrations in the 16- and 48-week-old control mice were not significantly different. In the ob/ob, db/db and HFD-fed mice, the insulin levels were increased by 10-, 9.5- and 5-fold in the 16-week-old animals (*P* = 0.002) and by 11.4-, 5.6- and 17.7-fold in the 48-week-old mice (*P* ≤ 0.004), respectively, as compared to the relevant controls. The overnight fasting resulted in a significant decrease in the insulin concentrations by 10.4- and 11.4-fold, respectively, in the young and old control mice (*P* ≤ 0.043), by 2.3- and 4.4-fold in the ob/ob mice (*P* ≤ 0.004), and by 4.8- and 11.6-fold in the HFD-fed mice (*P* = 0.004), as well as the not significant decreases of 1.7- and 1.6-fold in the db/db mice.

#### Liver function tests and serum lipid concentrations

The serum baseline ALT and AST levels were significantly increased in the 16-week-old ob/ob and db/db mice (*P* ≤ 0.005) and in all three groups of old obese mice (ALT, *P* ≤ 0.004; AST, *P* ≤ 0.009) compared to the respective controls. The serum level of ALKP was significantly higher in both the young and old ob/ob and db/db mice (*P* ≤ 0.005). While overnight fasting resulted in further increases in the aminotransferase levels in all three groups of old obese mice, starvation did not have any effect on the ALKP activity. Both 16- and 48-week-old obese mice developed hypercholesterolaemia (*P* ≤ 0.008), and the cholesterol concentrations were not affected by overnight fasting. Additionally, the serum levels of triglycerides were not affected by obese state.

Unsupervised hierarchical clustering revealed that the biochemical tests and the liver mass measurements clearly distinguished all groups of obese mice with hepatic steatosis from all control mice and young HFD-fed mice with histologically normal liver tissue, independent of fasting status (Fig. S2). However, in spite of the gradual alterations in most of the biochemical marker levels, these markers did not differentiate the obese mice with SS from those with NASH, which is in agreement with previous observations, indicating that the typical serum biomarkers associated with liver dysfunction, such as ALT or AST, are of low specificity and sensitivity in the diagnosis of NASH [35].

### Microarray-based selection of differentially expressed probe sets

To search for genes that were differentially expressed during the progression to NASH, mRNA from hepatic tissues was analysed by using the MouseRef-8 v2.0 Expression BeadChip microarray. In total, 48 microarrays containing 25,697 probe sets were hybridized. Of these, 10,755 probe sets passed our filtration criterion (IQR/median >1/8).

Principal component analysis decomposition of the microarray data sets of all of the liver samples revealed that overnight fasting was one of the strongest factors influencing the gene expression profile (data not shown), though further probe set selection was separately performed in the groups of non-fasting and fasting mice. To investigate the general transcriptional profiling related to SS and NASH, regardless of the mode of obesity induction, the animals were grouped based on the liver histological estimations into one of three groups: (1) control - represented by both young and old control mice and young wt HFD-fed mice, with normal liver histology; (2) SS – composed of young ob/ob and db/db mice with liver fat accumulation, without inflammation; and (3) NASH – consisted of old ob/ob, db/db and HFD-fed mice.

**Fig. 1:**
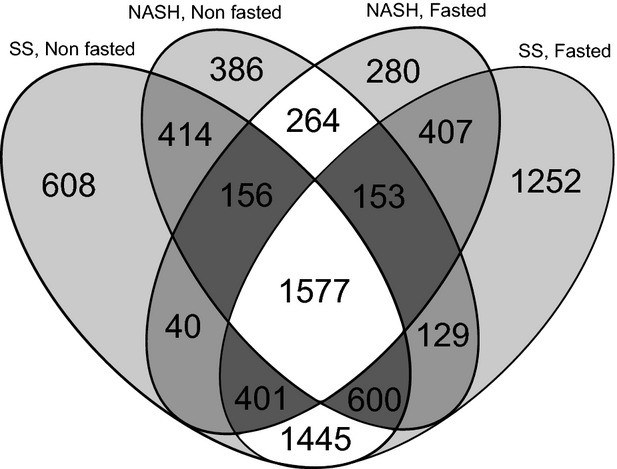
Venn diagram representing the common and unique differentially expressed probe sets in the simple steatosis (SS) and steatohepatitis (NASH) tissues. Combined comparison of the differentially expressed probe sets in fasted and non-fasted mice; a set of 1445 probes represented the genes altered exclusively in SS, a set of 264 probes represented the genes altered exclusively in NASH, and a set of 1577 probes represented the genes commonly changed in both SS and NASH.

In the pair-wise comparisons with the controls, we identified 5241/5964 and 3679/3278 probe sets that were differentially expressed (*P*_*adj*_ ≤ 0.05) in the non-fasted/fasted mice with SS and NASH, respectively (see Table S3 for all data sets). An additional 1414/1911 probe sets exhibited expression differences between the NASH and SS mice. Next, to define the global transcriptome changes that were not affected by metabolic stress related to prolonged fasting, the differentially expressed probe sets that commonly changed in the non-fasted and fasted mice were selected. These probe sets represented 4023 and 2150 probe sets in the mice with SS and NASH, respectively. Part of these changes, represented by 1577 probe sets, were common in both SS and NASH and, as such, could be considered prolonged alterations during the course of NAFLD progression (Fig. 1 and Table S3). In contrast, the levels of 1445 probe sets were changed exclusively in SS and 264 probes sets were altered in only NASH, representing the early and late transcriptional alterations during the progression of NAFLD, respectively.

### Functional annotations of the differentially expressed probe sets

To identify the fatty liver-relevant processes that were altered during the progression from normal tissue through SS to NASH, the signatures of the differentially expressed sets of 1445, 1577 and 264 probes (Fig. 1) representing the early, prolonged and late transcriptional alterations, respectively, were included in the interaction- and pathway-based analyses by using the CPDB database, which integrates different types of interactions from numerous resources into a seamless global network [30].

Several significantly overrepresented interactions and pathways were selected by CPDB analysis based on 1445 differentially expressed probe sets, exclusively reflecting early stage alterations leading to SS. These probe sets could be grouped into eight functional enriched pathways-based categories: *Mitochondria and energy metabolism*, *Translation*, *Transcription*, *Gut microbiota-related*, *Metabolism*, *Cytoskeleton and cellular transport*, *Cell proliferation and movement*, and *Neurological disorders* (Table S4). By using the 1577 probe sets that were commonly changed in both SS and NASH phenotypes, seven enriched pathways-based sets were selected: *ECM degradation*, *Metabolism*, *Coagulation and complement cascade*, *Nervous system*, *Cytoskeleton and cellular transport*, *Protein modification*, and *Cell proliferation and differentiation*. Finally, by using the 246 probe sets that differentiated the late stage of progression to NASH, only four pathways were uncovered: *Integrin*, *ECM organization*, *Collagen formation* and *Protein digestion and absorption*; three of these pathways are related to ECM organization and the cell-ECM interaction. It is worth noting that, although both prolonged and exclusively late stage transcriptome alterations involved ECM-related processes, these processes affected different types of ECM structures. Specifically, the initial ECM degradation processes, including the activation of the matrix metalloproteinases (MMPs), are followed by the organization of new ECM because of the formation of collagen fibres in the late, NASH-related stage.

### Differentially expressed genes that are consequently altered during the progression to NASH

We assumed that the genes that were found consequently altered during the progression from normal liver to SS and finally NASH, in concordance with the development of obesity, might be useful as molecular markers of NASH development. We search for such markers among the 1577 probe sets that were commonly altered in both SS and NASH, as compared to normal liver tissue. From these probe sets, 932 were up-regulated and 645 were down-regulated, and these probe sets represented 1302 genes (Table S3). Expression of 93 probe sets (representing 80 genes) from a set of 1577 was significantly changed in all three comparisons: SS *versus* control, NASH *versus* control and NASH *versus* SS (Table S5). Surprisingly, the expression of only three genes, which encoded epithelial membrane protein 1 (*Emp1*), IKBKB interacting protein (*Ikbip*) and decorin (*Dcn*), was consequently up-regulated during NASH progression, with *Dcn* showing an over threefold change in the NASH samples compared to normal liver. The remaining 77 genes exhibited alternating expression changes in the two types of tissue and were either up- or down-regulated during the progression from normal tissue to SS and oppositely down- or up-regulated during the progression from SS to NASH; none of the genes were consequently down-regulated.

**Fig. 2:**
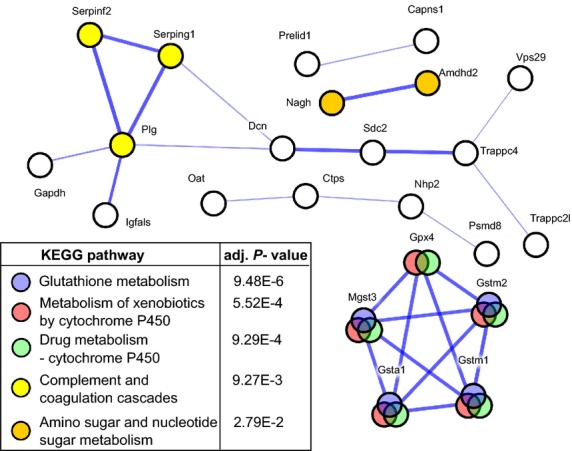
STRING interaction networks for a set of 80 proteins. The expression of genes encoded these proteins was significantly changed both in the simple steatosis and steatohepatitis tissues, as compared to normal liver tissue, and between these two types of tissues. The thicker network edges represent stronger evidence of association. The node colour depicts the significantly enriched KEGG category assigned to the set of 80 proteins by using the STRING database, while white nodes indicate proteins with other functions or without functional annotations.

The molecular context of the protein interactions encoded by these 80 genes was next explored by using the STRING database. Analysis revealed that these genes are highly interconnected (protein-protein interactions *P* = 4.89E-9) and significantly enriched for KEGG pathways involved in metabolism by cytochrome P450 (two pathways; Fig. 2). Other enriched KEGG pathways included *Glutathione metabolism*, *Complement and coagulation cascades*, and *Amino sugar and nucleotide sugar metabolism*. According to STRING, two main association networks were constructed by these proteins. Network 1 consisted of 10 proteins with Dcn, plasminogen (Plg) and trafficking protein particle complex 4 (Trappc4) located in the centre (Fig. 2). Network 2 consisted of five proteins that show well documented associations and are involved in *Glutathione metabolism* and *Metabolism of xenobiotics by cytochrome P450* pathways. This network included four members of the glutathione S-transferases (GSTs) family (Gsta1, Gstm1, Gstm2 and Mgst3) and glutathione peroxidase 4 (Gpx4). Of note, *Gsta1* showed the third highest expression change (over 11-fold) between SS and normal liver tissue.

### qRT-PCR verification of selected gene expression

**Fig. 3:**
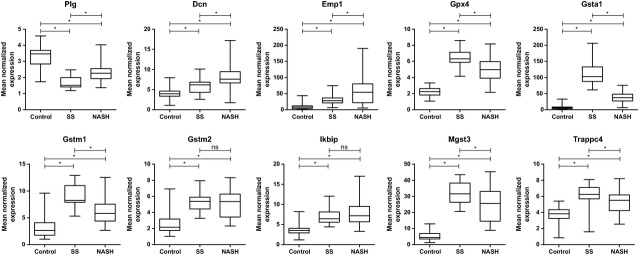
qRT-PCR analysis of 10 selected genes in 90 individual liver samples representing normal tissue (control; *n* = 36), simple steatosis (SS; *n* = 21) and steatohepatitis (NASH; *n* = 33). One microgram of total RNA was reverse-transcribed to generate cDNA, and q-PCR was performed with SYBR Green I chemistry. The box border represents the interquartile range and the horizontal line in the box represents the median. The whiskers denote the largest/smallest observation. The statistical significance of the differences was assessed by using a *t*-test and was corrected for multiple hypotheses testing by using the Benjamini-Hochberg procedure. (*) Adjusted *P*-values ≤ 0.05 were considered as significant. ns, not significant.

We selected 10 genes for further expression verification by using qRT-PCR. These genes included the three central genes from Network 1 (*Plg*, *Dcn* and *Trappc4*), all five genes from Network 2 and additional two genes that were consequently up-regulated during the progression to NASH (*Ikbip*, *Emp1*). With the exception of two genes in NASH *versus* SS comparison, all of these genes showed highly significant (*P*_*adj*_ ≤ 0.05) progressive changes in expression during the course of NASH development (Fig. 3, Table S6). According to the microarray analysis, *Gsta1* exhibited the highest expression change between SS and normal tissue (FC > 16.24) and *Emp1* in the progression of SS to NASH (FC > 1.95).

**Fig. 4:**
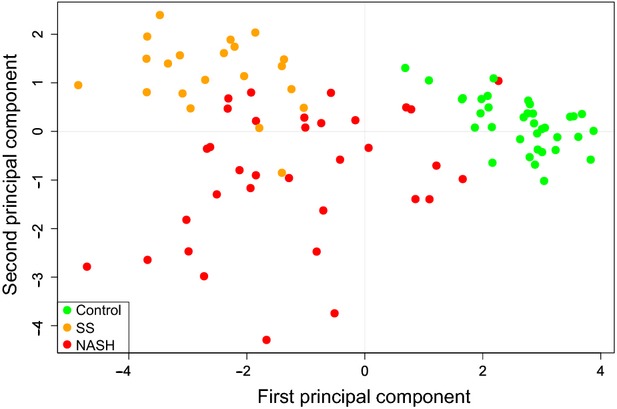
Principal component analysis decomposition of the qRT-PCR data sets for liver samples representing normal tissue (control), simple steatosis (SS) and steatohepatitis (NASH).

Principal component analysis decomposition of the qRT-PCR data sets for normal liver, SS and NASH tissue samples revealed that the expression profile of these 10 selected genes almost perfectly distinguished the hepatic tissue with NASH from both the normal tissue and those with SS (Fig. 4).

## Discussion

A comparative analysis of the results from transcriptional-based studies conducted over the past decade in obese humans or rodents revealed several dozen to thousands of differentially expressed genes in NAFLD samples, depending on the experimental conditions and the statistics included in the data processing. Consequently, numerous altered liver functional networks have been reported, including mitochondrial dysfunction, inflammation, ECM remodelling, apoptosis, hepatic detoxification, liver regeneration and fibrosis, to name a few [10,16–18,36–41]. However, the complex interplay between these molecular alterations during the progression of fatty liver to NASH is still poorly understood.

Our microarray-based study focused on identifying the general gene expression alterations that are relevant to NASH progression and are independent of the mode of obesity induction and feeding condition. Therefore, animals from three obesity models (ob/ob, db/db and HFD-fed C57BL/6J) and ND-fed C57BL/6J control mice were combined, based on their histological characteristics, into three groups that represented normal liver, SS, and NASH. Of them, mice with fatty liver were additionally distinguished from mice with histologically normal livers because of their altered serum biochemical measures (Fig. S2), such as hyperinsulinaemia, hyperglycaemia and hypercholesterolaemia, as well as increased ALT, AST and ALKP activities (Table 1). As we recently published [33], these mice were also characterized by several alterations related to lipid and glucose homoeostasis, which was shown based on mitochondrial-related proteomic changes. Altogether, the mice with liver steatosis in the studied obesity models reproduced the key features of NAFLD in humans, including the progression to NASH [1,7].

We selected differentially expressed probe sets that represented the early stage of NAFLD development (characteristic for liver with SS only), the late stage of changes (characteristic exclusively for NASH) and prolonged alterations (common for both SS and NASH). The intersections of the differentiated probe sets, which were not affected by a fasting state, were further functionally annotated. The molecular and cellular pathways that described multiple functional aspects of fatty liver development and its progression to NASH were extracted. Most of these pathways were related to the early stage of fatty liver, and they represented several metabolic processes, in particular those connected to energy metabolism and mitochondria, as well as the translation and transcription processes (Table S4). In contrast, only four pathways, including three related to ECM organization, were characteristic for the late stage, which was equivalent to NASH. Thus, contrary to the more profound molecular changes in human NASH, compared to SS, reported by Starmann *et al*. [10], our study indicates more complex interplay between the molecular alterations in the early stages of NAFLD development. Such a difference may result from the lack of true control tissues that represent histologically normal livers in Starmann *et al*. study, which is an obvious disadvantage of human studies.

When fatty liver progresses, the deregulation of processes involved in other functional categories predominate over the energetic and metabolic pathways. Consistently, the current understanding of human NAFLD pathogenesis is based on a ‘two-hit’ model, in which the ‘first hit’ relates to the accumulation of intracellular lipids leading to metabolic and molecular alterations that sensitizes the liver to the induction of inflammation, and the ‘second hit’ promotes the excess production of reactive oxygen species (ROS), which are required for the advancement to NASH [42]. Oxidative stress and the enhanced production of ROS in fatty liver lead to hepatic apoptosis and necrosis [14,19,43]. This complex process begins with monocyte migration across the endothelium and their differentiation into tissue macrophages [44], which secrete the pro-inflammatory cytokines TNF-α and IL-6, further enhancing the monocyte influx and the decrease in insulin sensitivity [45]. Accordingly, our proteomic study [33] indicated the deregulation of proteins involved in the *Leukocyte transendothelial migration*, *Tight junction* and *Regulation of actin cytoskeleton* KEGG pathways in obese mice with NASH. Thus, both global chronic inflammation and local liver injury can promote further morphological and functional hepatic alterations, which are hallmarks of NASH [14,18,19,21].

In this study, ECM-related annotations were identified for the differentially expressed genes associated with both prolonged and late stage changes of NASH progression (Table S4). However, while the ECM degradation processes related to MMP activation were common for SS and NASH, ECM organization with collagen fibre production and ECM-cell interaction *via* integrins were exclusively identified in the NASH samples. Similarly, the up-regulation of genes associated with molecular processes typical for tumour progression, such as cell adhesion, cell movement, integrin signalling and ECM, was recently shown to be characteristic of NASH in humans, although the SS and control tissues were combined in that study [10].

In search for markers of the progression to NASH, we uncovered 80 genes that had significantly altered expression in both SS and NASH, as compared with normal liver tissue, and between SS and NASH (Table S5). Three of these genes (*Dcn*, *Emp1* and *Ikbip*) were consequently up-regulated in progression from normal tissue, through SS to NASH.

Decorin is a small leucine-rich proteoglycan that is ubiquitous in the ECM of different tissues. This protein may influence ECM architecture and stabilize the inter-fibrilar organization by binding to the collagen fibrils [46,47], as well as modulate the other ECM components binding to the cells *via* integrins [48] and attenuate transforming growth factor (TGF)-β1 activity [49]. Decorin binding to TGF-β1 prevents its interaction with pro-fibrotic receptors, such as EGFR, IGFR and Met [50], and the relevant enhancement of downstream Erk1/2 activation was observed in *Dcn*-null mice [51]. Decorin is directly involved in maintaining the balance between matrix synthesis and degradation, as fibrotic livers in the *Dcn-*null mice were characterized by low activity of MMP-2 and MMP-9 and high expression of the tissue inhibitor of MMPs (TIMP)-1 [51]. Decorin is produced by monocytes and macrophages at sites of inflammation [52]. Furthermore, its expression in adipose tissue was markedly up-regulated in the obese state [53]. In our study, decorin expression was gradually increased during the progression to NASH (Table S5), suggesting a stimulatory effect on the development of inflammation.

The expression of the *Emp1* gene was increased over threefold in NASH compared to normal tissue and by 1.8-fold compared to SS tissue; being one of the most highly up-regulated genes in the NASH tissues (Table S5). Emp1 is a member of the peripheral myelin protein (PMP22) family and has been described as a putative tight junction protein in a liver stem cell line and in the liver [54]. The other member of this family, Emp2, regulates the surface expression of specific integrin isoforms [55] and modulates collagen gel contraction in a process dependent on enhanced FAK activation [55]. Altogether, these observations suggest that the Emp proteins may modulate cell-matrix and cell-cell interactions, thus influencing processes involving membrane trafficking, cell adhesion and migration.

Third gene that was consequently up-regulated during the progression to NASH (Table S5), *Ikbip*, encodes for IκB kinase β (IKBKB) interacting protein (IKIP) of endoplasmic reticulum (ER) localization. This gene is highly conserved and links the transcription factor NF-κB to the apoptotic signalling pathway in the ER [56]. Increased transcriptional activity of NF-κB was observed in the livers of obese mice, which were induced by genetic hyperphagia or a HFD [2]. Deletion of IKBKB reduces cytokine expression, including IL-6 and TNF-α, as well as subacute hepatic inflammation, thus protecting against local hepatic and global IR [57].

The molecular context analysis of the proteins encoded by 80 selected genes that were commonly altered in both SS and NASH and between these two stages revealed that they are significantly enriched in five KEGG pathways. Further protein interaction analysis showed that 15 of these proteins are interconnected into two main association networks (Fig. 2). Network 1 consisted of 10 proteins related to membrane trafficking and the complement and coagulation cascade with centrally located Dcn. Network 2 contained five proteins involved in glutathione and drug metabolism, including four GST family members (Gsta1, Gstm1, Gstm2, Mgst3) and Gpx4. Among the genes encoding these proteins, *Gsta1* showed an over 11-fold increase in expression in the SS tissues; although this level significantly decreased during the progression from SS to NASH, its expression remained elevated in NASH when compared to normal liver. Similarly, *Gsta1* was significantly overexpressed in the ob/ob mice as compared to the wt C57BL/6J [41]. It is suggested that, although the increased expression of *Gsta1* protects against oxidative stress, the down-regulation of *Gsta1* during chronic inflammation, such as that related to obesity, may potentiate its cytotoxic effects [58–61].

In conclusion, our studies performed with different mouse models of obesity induced by genetic hyperphagia or a HFD confirmed the involvement of ECM-related processes in the progression to NASH, which was recently established in human fatty liver disease [10]. We described three genes (*Dcn*, *Emp1* and *Ikbip*), the expression levels of which were consequently up-regulated during the progression from normal liver tissue through SS to NASH. Two of these genes are involved in ECM-cell or cell-cell interactions. The expression profile of 10 selected differentially expressed genes along the course of NASH development has the potential to distinguish NASH livers from SS and normal tissues and, as such, may be beneficial in the clinical diagnosis of NASH.

## Supporting information

**Figure S1**. Representative photographs of liver tissues stained with haematoxylin and eosin (magnification, 200×).

**Figure S2**. Heat-map of unsupervised hierarchical clustering analysis of the different mice according to the blood biochemical measures and liver weight.

**Table S1**. Histological characteristics of liver tissues. **Table S2**. Primer pairs used for qRT-PCR. **Table S3**. Data sets of the pair-wise comparisons of the levels of all microarray probe sets. **Table S4**. Significant enriched pathway-based sets according to the ConsensusPathDB interaction analysis of the relevant differentially expressed probe sets. **Table S5**. Differentially expressed probe sets along the course of the progression to NASH. **Table S6**. Expression profiles of 10 selected genes according to the qRT-PCR analysis.
