# Supplementary figures and images for: Extracellular matrix and cytochrome P450 gene expression can distinguish steatohepatitis from steatosis in mice
Source: J Cell Mol Med. 2014 Jun 9;18(9):1762–72. doi: 10.1111/jcmm.12328 (PMC4196652; doi:10.1111/jcmm.12328)

**A**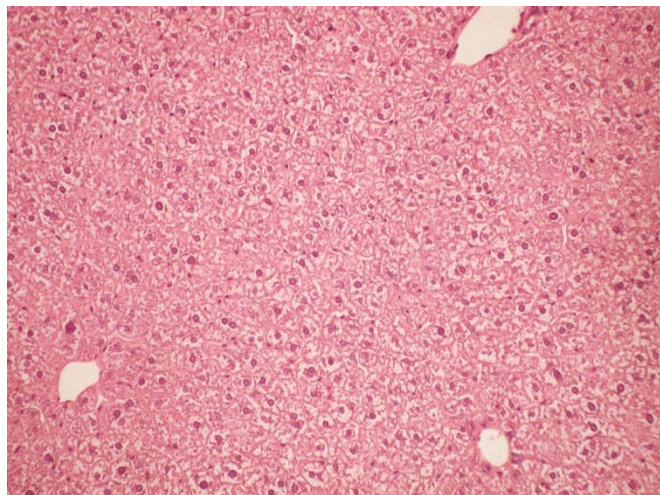**B**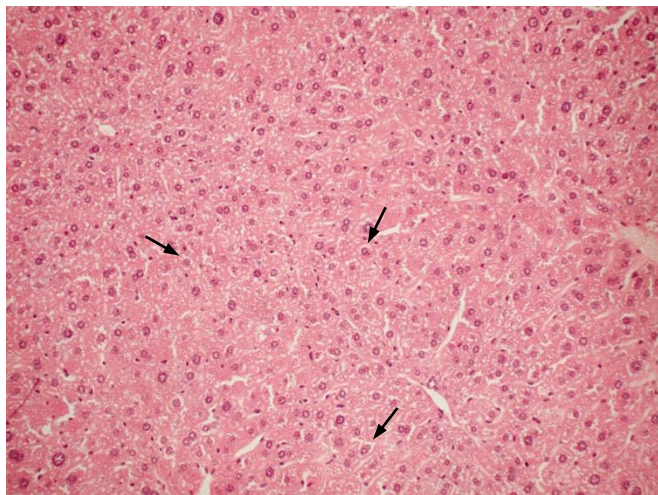**C**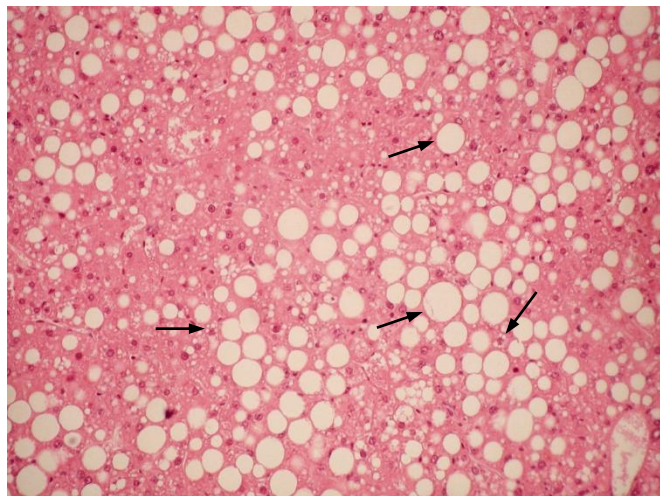**D**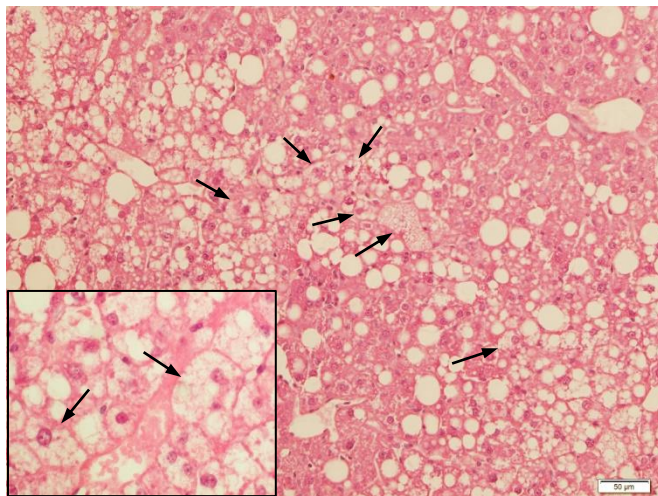**E**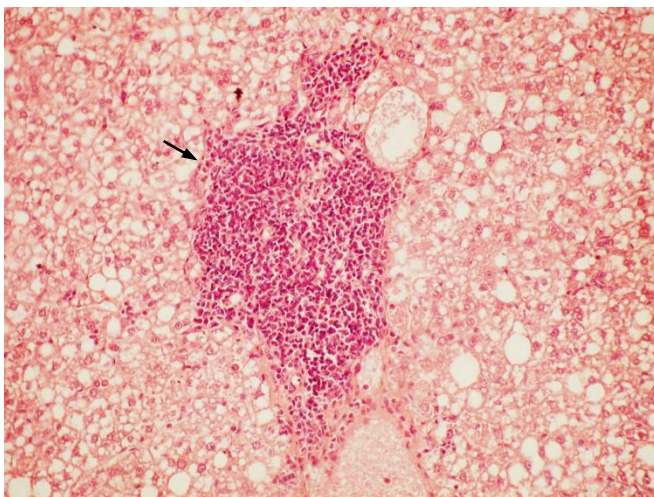

Supplement: Supplementary file 1 — Figure S1. Representative photographs of liver tissues stained with haematoxylin and eosin (magnification, 200×). [file jcmm0018-1762-SD1.pdf]

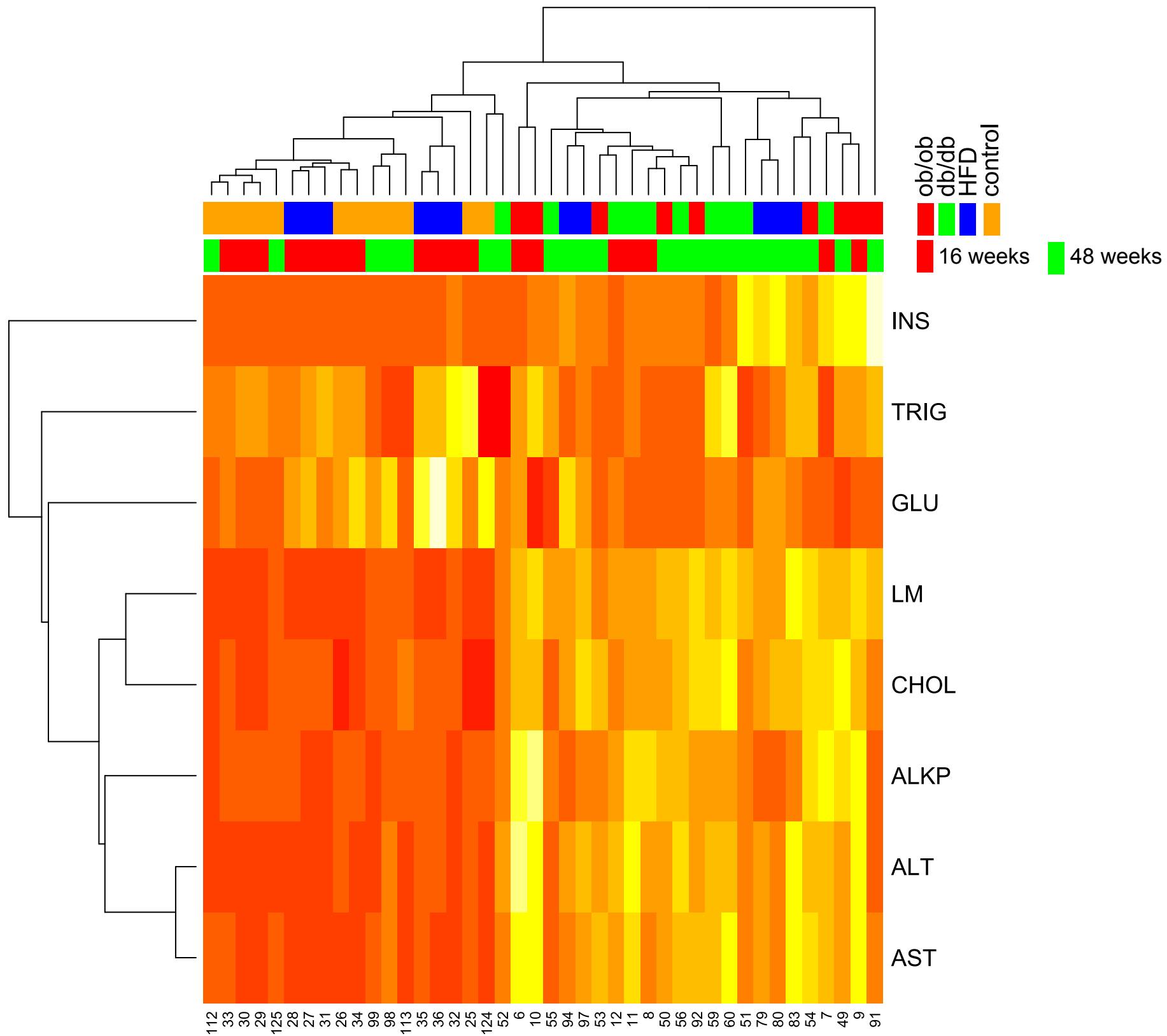

Supplement: Supplementary file 2 — Figure S2. Heat-map of unsupervised hierarchical clustering analysis of the different mice according to the blood biochemical measures and liver weight. [file jcmm0018-1762-SD2.pdf]
